# Adsorption Behavior of Polymer Chain with Different Topology Structure at the Polymer-Nanoparticle Interface

**DOI:** 10.3390/polym10060590

**Published:** 2018-05-28

**Authors:** Qingliang Song, Yongyun Ji, Shiben Li, Xianghong Wang, Linli He

**Affiliations:** Department of Physics, Wenzhou University, Wenzhou 325035, China; qingliangsong1992@163.com (Q.S.); yyji@wzu.edu.cn (Y.J.); shibenli@wzu.edu.cn (S.L.); wxh@wzvtc.edu.cn (X.W.)

**Keywords:** chain topology, selective adsorption, polymer-NP interface

## Abstract

The effect of the polymer chain topology structure on the adsorption behavior in the polymer-nanoparticle (NP) interface is investigated by employing coarse-grained molecular dynamics simulations in various polymer-NP interaction and chain stiffness. At a weak polymer-NP interaction, ring chain with a closed topology structure has a slight priority to occupy the interfacial region than linear chain. At a strong polymer-NP interaction, the “middle” adsorption mechanism dominates the polymer local packing in the interface. As the increase of chain stiffness, an interesting transition from ring to linear chain preferential adsorption behavior occurs. The semiflexible linear chain squeezes ring chain out of the interfacial region by forming a helical structure and wrapping tightly the surface of NP. In particular, this selective adsorption behavior becomes more dramatic for the case of rigid-like chain, in which 3D tangent conformation of linear chain is absolutely prior to the 2D plane orbital structure of ring chain. The local packing and competitive adsorption behavior of bidisperse matrix in polymer-NP interface can be explained based on the adsorption mechanism of monodisperse (pure ring or linear) case. These investigations may provide some insights into polymer-NP interfacial adsorption behavior and guide the design of high-performance nanocomposites.

## 1. Introduction

Polymer nanocomposites that consist of mixtures of polymers and organic/inorganic particles are a new member of composite materials, which have been used in a wide variety fields [1]. The macroscopic properties (such as mechanic, electronic, optical, and so forth) of nanocomposites not only depend on the microscopic morphology of constituent nanoparticle (NP) in the polymer matrix [2,3,4,5,6], but also are sensitive to the polymer conformation, especially the local packing at polymer-NP interface. For example, carbon black particles are immersed to increase the strength, viscosity, and durability of rubbers [7,8], and fullerenes are used to enhance the efficiency of polymer-based photovoltaic devices [9].

Nevertheless, it has been proven that the adding of particles into polymeric materials frequently results in agglomeration and phase separation, a uniform dispersion of NPs in polymer matrix is hard to get, owing to the strong interparticle interactions and weak polymer-NP interfacial interaction. Many experimental [10,11], theoretical [12,13], and simulation [14,15,16,17] studies have been devoted to investigating this issue. Hooper et al. theoretically pointed out that there are four general categories of polymer-mediated NP organization: contact aggregation due to depletion attraction, segment level tight particle bridging, steric stabilization due to thermodynamically stable “bound polymer layers”, and “tele-bridging” where distinct adsorbed layers coexist with longer range bridging [12]. Mackay et al. experimentally demonstrated that thermodynamically stable dispersion of NPs into a polymeric liquid is enhanced when the radius of gyration of the linear polymer is greater than the NP radius [10]. Liu et al. emphasized that a homogeneous filler dispersion exists just at the intermediate interfacial interaction by computer simulations [14]. In a subsequent study by Singh et al., they analyzed the transitions between different structures in the polymer-NP system caused by the polymer-NP interaction strength and polymer chain length, respectively [15].

On the other hand, some researchers have paid their attention to the polymer conformation behavior in nanocomposites, such as the dimension [11,16,18,19,20]. However, up to date, there is also controversy as to whether the addition of NPs to a polymer melt causes polymer chains either to expand, shrink or be unperturbed compared to their size in the bulk. For instance, Mackay et al. found that when the radius of gyration of the polymer is larger than the chemically identical NP radius, a 10–20% expansion in deuterated polystyrene (d-PS) chain dimensions occurs by neutron scattering [11]. However, Crawford et al. ensured a spatially uniform dispersion of 13 nm silica NPs miscible in polystyrene melts by neutron scattering, X-ray scattering, and transmission electron microscopy. They found that the polymer size in nanocomposites remains unaltered regardless of the relative size between components and NP loading [16]. From a simulation point of view, Clarke et al. demonstrated that the polymer chains are unperturbed by the presence of repulsive NPs, while it can be stretched and flattened by the attractive NPs with very small size [20]. Yan et al. reviewed the recent progress on the structure, dynamics, and physical properties of PNCs [21]. Besides the investigation on the NP dispersion and polymer conformation, some attentions are also drawn to understanding the local packing [14,22,23,24,25] and dynamics [26,27,28,29] of polymer matrix at the polymer-NP interface. Chen et al. [22] experimentally observed double glass transition and interfacial immobilized layer in in-Situ-Synthesized PVA/Silica Nanocomposites, and pointed out the interfacial layer is mainly composed of partial segments of different polymer chains, which is further verified by the molecular dynamics simulation of Liu et al. [14]. Vacatello et al. used Monte Carlo (MC) simulations to obtain a general picture of the molecular arrangements in polymer-based nanocomposites, in which the chains are classified as the sequences of interface, bridge and loop segments [24]. Ge et al. [26] investigated the role of polymer topology in the dynamical coupling between NPs and polymers in nanocomposites. Kumar et al. [27] studied the diffusion of nanoparticle in polymer nanocomposites and provided an excellent understanding on the motion of NPs, which the size is smaller than the polymer entanglement mesh size. Besides, Ying et al. [28,29] used molecular dynamics simulation to study the effect of nanoparticle volume fraction on the dynamics of polymer and offered an explanation for understanding the rheological properties of polymer composites.

Obviously, polymer chains around NP exhibit dramatically different interfacial behavior in comparison with the bulk phase. Several groups [30,31,32] paid their attention on the adsorption mechanism of polymers on flat surfaces. For instance, Cohen-Stuart et al. [30] performed MC simulations for studying the competition between surface adsorption and folding of fibril-forming polypeptides, and their results suggest that a weakly attractive surface can enhance the folding. Sommer et al. [31] used computer simulations on the adsorption of branched and dendritic polymers onto flat surfaces, there is a two-step adsorption scenario on temperature dependence related with strong excluded volume effects.

Nevertheless, there is no general experimental, theoretical, and simulation consensus about the accurate characterization and interpretation of the interfacial region, especially considering the adsorption mechanism of polymer chains with polydispersity close to the surface of NP. We have involved this issue by exploring the interfacial adsorption mechanism by varying the polymer-NP interaction, which may be the main factor in determining the interfacial behavior. Our study further confirmed that one of chain ends prefers to be in contact with NP and shows a perpendicular conformation to the NP surface when the polymer-NP interactions are weak (i.e., endpoint adsorption); while the inner chain monomers wrap NP tightly when the attractive interactions are strong (i.e., middle adsorption) [25]. In order to further characterize the adsorption mechanism and local packing of bidisperse matrix with different topologies in the polymer-NP interfacial region, we focus on the mixtures of ring and linear polymer chain in nanocomposites.

## 2. Model and Methods

In our simulation, a standard bead-spring model is used to model polymer chain. Each ring or linear polymer chain consists of *n* spherical monomers with diameter of σ and mass of *m*, which are interconnected by the finitely extendable nonlinear elastic (FENE) potential [33]:

(1) $$U_{\mathrm{FENE}}(r)=-\frac{KR_{0}^{2}}{2}\ln\left[1-{\left(\frac{r}{R_{0}}\right)}^{2}\right],r<R_{0}$$

where *r* is the distance between the two neighboring monomers. $K=30\varepsilon /\sigma^{2}$ is a spring constant and *R*_0_ = 1.5σ is a finite extensibility to avoid chain crossing, where σ is the monomer diameter. The stiffness of a polymer chain is described by a bending potential between adjacent bonds:

(2) $$U_{\mathrm{bending}}=k_{b}(1+\cos\theta )$$

where θ is the angle between two consecutive bonds and the chain stiffness is controlled by varying the value of $k_{b}$. NP is modeled as a Lennard-Jones (LJ) sphere of radius *R*_n_ = 2.5σ. Mass density of NPs are the same as the polymers, therefore the mass of NP is 125 times of that of a monomer.

Here a truncated and shifted Lennard-Jones (LJ) potential is used to model NP-NP and polymer-NP interactions as well as the nonbonded interactions between all polymer monomers, as follows [34]:

(3) $$U\left(r\right)=\left\{ \begin{array}{ll} 4\varepsilon \left[{\left(\frac{\sigma }{r-r_{\mathrm{EV}}}\right)}^{12}-{\left(\frac{\sigma }{r-r_{\mathrm{EV}}}\right)}^{6}+\frac{1}{4}\right] & r-r_{\mathrm{EV}}<r_{\mathrm{cutoff}}\\ 0 & r-r_{\mathrm{EV}}\geq r_{\mathrm{cutoff}} \end{array}\right.$$

where ε is the LJ potential interaction energy and $r_{\mathrm{cutoff}}$ stands for the distance $\left(r-r_{\mathrm{EV}}\right)$ at which the interaction is truncated and shifted so that the energy and force are zero. In our simulation, we offset the interaction range by $r_{\mathrm{EV}}$ to account for the excluded volume effects of different interaction sites. For NP-NP and polymer-NP interactions, $r_{\mathrm{EV}}$ equals to $2R_{n}-\sigma$ and $R_{n}-\sigma /2$, respectively, while for polymer-polymer interactions, $r_{\mathrm{EV}}$ is zero. Here $\varepsilon_{\mathrm{nn}}=\varepsilon_{\mathrm{pp}}=1.0$ and $r_{\mathrm{cutoff}}=2^{1/6}\sigma$ in both NP-NP and polymer-polymer interactions with repulsive only part of Equation (3). Meantime, $\varepsilon_{\mathrm{np}}$ is varied to simulate different interfacial interactions with an attractive nature of $r_{\mathrm{cutoff}}=2.5\sigma$. Here we set ε and σ to be unity for dimensionless simulation.

Our molecular dynamics simulations are performed in a NVT ensemble. The simulation box is set as 35σ×35σ×35σ, where periodic boundary conditions are employed in all three directions. NPs with a fixed number of M = 5 are embedded at a random position and allowed to move. Here the polymer bulk density is set as $\rho^{*}=\;0.8$ and each polymer chain consists of *n* = 30 monomers. 570 ring chains with *n* = 30 and 570 linear chains with *n* = 30 are mixed in bidisperse case, while 1140 pure ring or linear chains with *n* = 30 are selected in monodisperse case. Additionally, the velocity-verlet algorithm is used to integrate the equation of motions with time step Δt=0.01τ, where the unit of time $\tau =\sqrt{\varepsilon /m\sigma^{2}}$ and m is the mass unit of a monomer. The desired temperature is set to T=1.0 by using a Langevin thermostat. Rapid annealing from the initial temperature T=9 to T=5 is initially employed, followed by a slow anneal-temper process between T=5 and the desired temperature is performed, to prevent the simulations from trapping into a local minimum energy at a low temperature. All simulations were performed by the open source LAMMPS molecular dynamics package [35].

## 3. Results and Discussion

To characterize the local packing and competitive adsorption mechanism of bidisperse matrix in the polymer-NP interfacial region, the nanocomposites with the mixture of ring and linear polymer chain are considered. We change the interaction strength, $\varepsilon_{\mathrm{np}}$, from 0.1 to 10.0, representing weak to strong polymer-NP attraction, and the chain stiffness, $k_{b}$, from 0 to 50, which corresponds to the flexible, semiflexible, and rigid-like chain, respectively. Then the monodisperse case (pure ring or linear chain) is also analyzed to interpret the intrinsic reason for the competitive adsorption occurred in bidisperse case.

**Figure 1:**
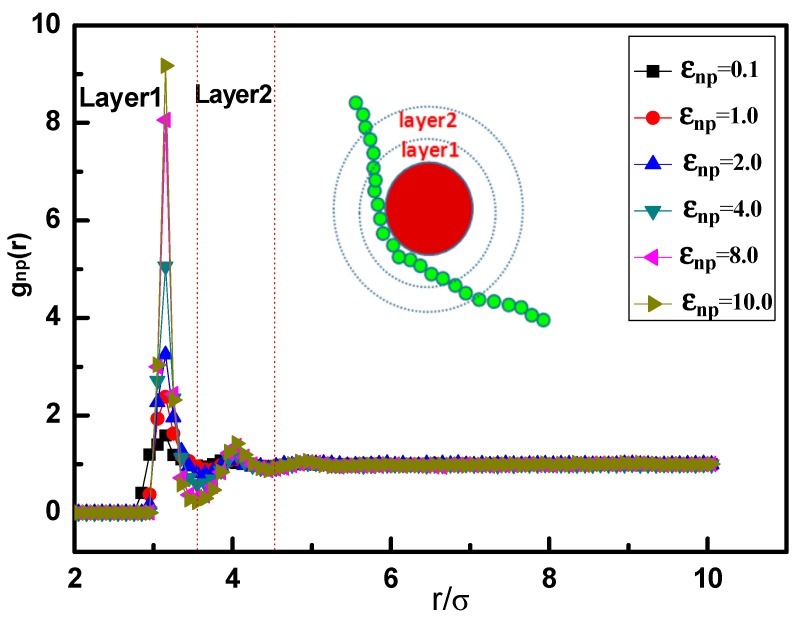
Polymer-nanoparticle (NP) pair distribution function $g_{\mathrm{np}}(r)$ in nanocomposites for various attractive interactions $\varepsilon_{\mathrm{np}}$.

Before the discussion, we first examine the local structure of polymer close to the surface of NP in nanocomposites and define the polymer-NP interfacial region. Take pure linear chain for example, as shown in Figure 1, the polymer-NP pair distribution function $g_{\mathrm{np}}(r)$ for various attraction $\varepsilon_{\mathrm{np}}$ exhibits an evident layering behavior. The obviously high monomer density in “Layer1” (shown in Figure 1) establishes a well defined interface between the polymer and NP, therefore we take the “Layer1” as the polymer-NP interfacial region in the following study. Meanwhile, we present the whole chain in the snapshots of nanocomposites by only one or more monomers per chain located within Layer1. Figure 1 also shows that the polymer density around NP increases with increasing $\varepsilon_{\mathrm{np}}$ from weak to strong attraction, which is consistent with the studies by Gao et al. [14] and Karatrantos et al. [20].

**Figure 2:**
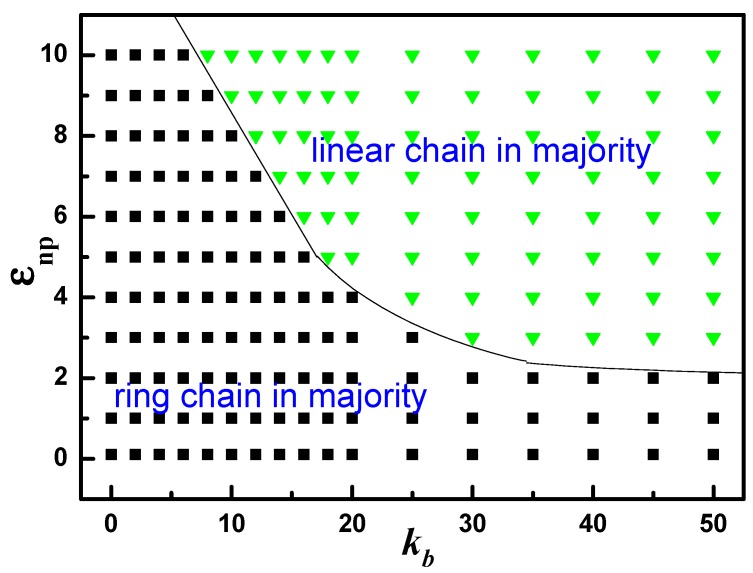
The phase diagram of ring and linear chains adsorbed to polymer-NP interfacial region as a function of polymer-NP interaction $\varepsilon_{\mathrm{np}}$ and chain stiffness $k_{b}$.

Then we focus on the bidisperse matrix of the mixture of ring and linear polymer chain with the same amount of monomer. Figure 2 represents an overview of the selective adsorption states that arise upon varying the two main variables, the polymer-NP interaction $\varepsilon_{\mathrm{np}}$ and chain stiffness $k_{b}$. Here “ring chain in majority” in phase diagram indicates that the number of ring chain monomers in polymer-NP interface is more than that of linear chain, while “linear chain in majority” is opposite. The solid line in Figure 2 is used to divided the above two adsorption states. In the case of weak and intermediate attractive interactions $\varepsilon_{\mathrm{np}}$ such as from 0.1 to 2.0, ring chain always takes precedence over the linear chain regardless of chain stiffness $k_{b}$. For the strong attractive interactions, $\varepsilon_{\mathrm{np}}>2.0$ there is a preferential transition from ‘ring chain in majority’ to ‘linear chain in majority’ as the chain stiffness $k_{b}$ increases. From figure, we also can see that the transition point decreases with the increase of $k_{b}$. This finding may be related to the different local packing of ring and linear semiflexible chain, which will be discussed detailly in the follow.

**Figure 3:**
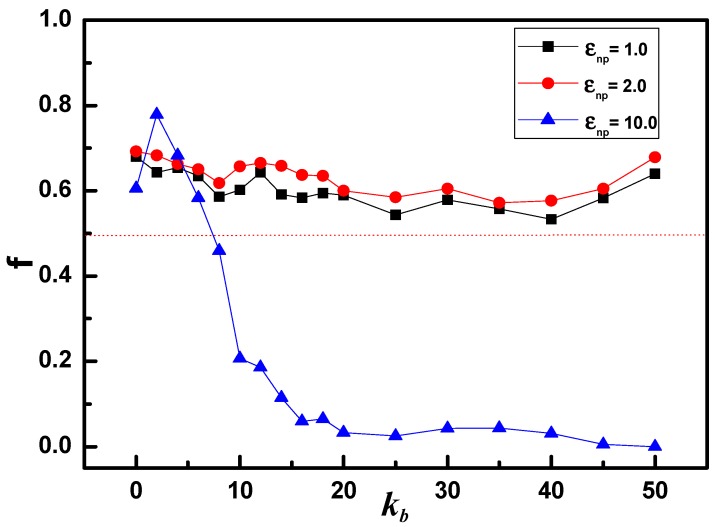
The fraction of ring chain to all chain in polymer-NP interfacial region, as a function of chain stiffness $k_{b}$ for three attractive interactions.

To further quantitatively understand the preferential adsorption behavior between ring chains and linear chains in interfacial region, the fractions of ring chain to all monomers in polymer-NP interface f for various interactions are calculated shown in Figure 3. As $\varepsilon_{\mathrm{np}}=\;1.0$ or 2.0, the value of f is in the range of 0.5 to 0.7, indicating that the ring chain slightly preferentially occupy the interfacial region. As f approximately equals to 0.5, means that the interfacial region shows no selection for ring or linear chains. At the strong polymer-NP interaction, $\varepsilon_{\mathrm{np}}=\;10.0$, f>0.5 as $0<k_{b}\leq 6$, indicating that the ring chain preferentially occupy the interfacial region. Interestingly, the value of f shifts down to be less than 0.5 as $k_{b}$ increases from 8 to 50. This result suggests the ring chains in interfacial region are quickly replaced by linear chains, as the chain becomes stiffer. The minimum value of f is even close to zero as $k_{b}\approx 50$, showing an absolute priority for linear chains to occupy the polymer-NP interface.

**Figure 4:**
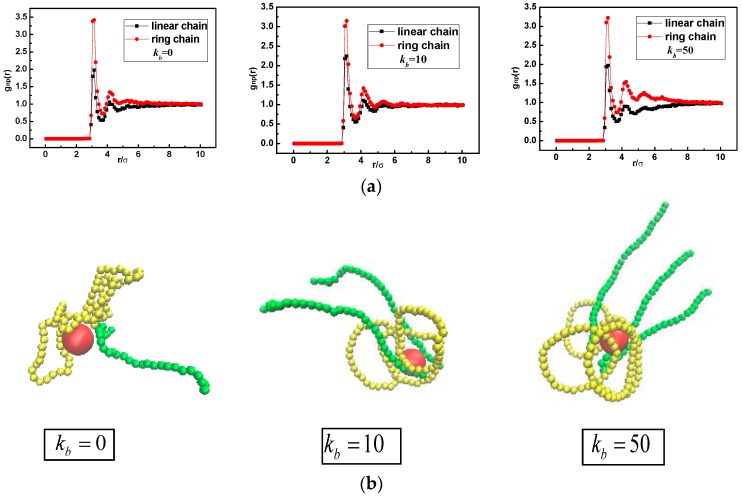
(**a**) Polymer-NP pair distribution function $g_{\mathrm{np}}(r)$; (**b**) snapshots of the conformations of nanocomposites with $\varepsilon_{\mathrm{np}}=\;1.0$. Here ring and linear polymer chain are displayed in yellow and green, respectively.

**Figure 5:**
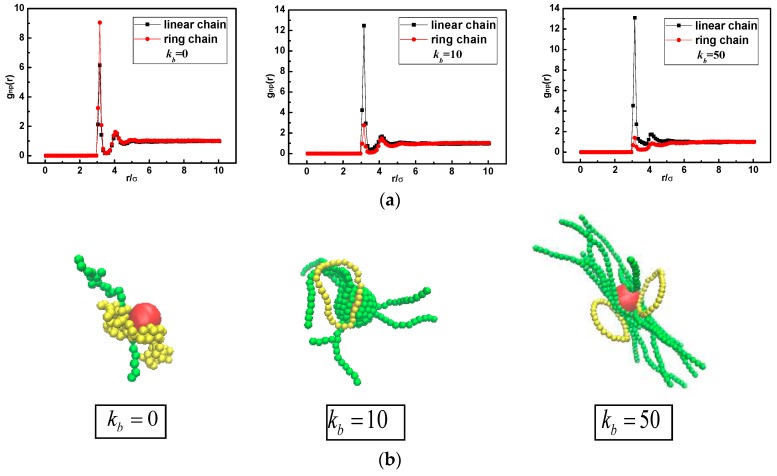
(**a**) Polymer-NP pair distribution function $g_{\mathrm{np}}(r)$; (**b**) snapshots of the conformations of nanocomposites with $\varepsilon_{\mathrm{np}}=\;10.0$.

Meanwhile the polymer-NP pair distribution function $g_{\mathrm{np}}(r)$ and its representative snapshots for bidisperse matrix are shown in Figure 4 and Figure 5, respectively. At the weak polymer-NP interactions $\varepsilon_{\mathrm{np}}=\;1.0$ for $k_{b}$ = 0, 10, and 50, snapshots in Figure 4b display that the interfacial layer is composed of partial segments of ring and linear polymer chains. Ring chains preferentially wet the interfacial layer more than linear chains, in which the peak in $g_{\mathrm{np}}(r)$ for ring chain is slightly higher than the peak for linear chain shown in Figure 4a. Similar behavior appears in nanocomposites regardless of the chain stiffness varied from flexible ($k_{b}$ = 0) to semiflexible ($k_{b}$ = 10) and to rigid-like ($k_{b}$ = 50) chain. Some related studies have pointed out that at a weak polymer-NP interaction, polymer chains prefer to performing a “one-endpoint” adsorption behavior, which shows a perpendicular conformation to the NP surface [14,25]. As a result, it can be referred that for weak polymer-NP interaction, ring chains show a higher correlation with NP due to its closed topology structure, and there is no evident chain stiffness dependence.

For the strong polymer-NP interactions $\varepsilon_{\mathrm{np}}=\;10.0$, our previous study also has given the results that the inner monomers of chains prefer to wrap tightly the surface of NP, which performs a “middle” adsorption behavior [25,36]. Figure 5 shows a ring-to-linear selective adsorption transition with increasing chain stiffness. As $k_{b}$ = 0, i.e., flexible chain, this “middle” adsorption mechanism that the middle monomers of polymer chain tend to cover the surface of NP, drives the ring chains still in majority in comparison with linear chain, due to its closed topology structure. As $k_{b}$ = 10, i.e., semiflexible chain, the middle image in Figure 5b shows that the linear chains favorably wrap around NPs and predominantly crowd the ring chains out of the interfacial region. On the one hand, this phenomenon can be attributed to the fact that the linear semiflexible chains with a proper stiffness are inclined to form a chiral helical structure [37], which is commonly observed in biological environments. For instance, the negatively charged stiff polymer (DNA) wraps around a cationic core particle (protein), in which the packing manner is referred to as a nucleosome-like structure [38,39,40].

On the other hand, for the ring chain, the entropy gain due to the possible number of states of the chain near to the surface dominates the bending energy cost. As a result, the semiflexible linear chain tends to occupy primarily the interfacial region. With increasing $k_{b}$ = 50, i.e., rigid-like chain, the preferential adsorption behavior by linear chains becomes more pronounced shown in the curve of $g_{\mathrm{np}}(r)$. The spiral linear chain begins to untie and tangentially cover the NP surface with the middle parts of the chain, and little interfacial region is left for the rigid ring, which corresponds to the adsorption state of f≈0 mentioned in Figure 3.

**Figure 6:**
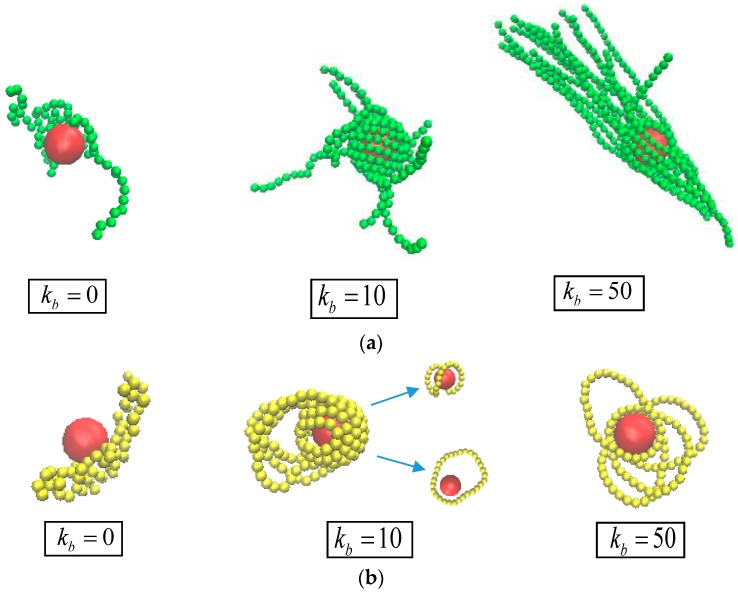
Snapshots of the conformations of nanocomposites at a strong attraction, $\varepsilon_{\mathrm{np}}=\;10.0$ with $k_{b}$ = 0, 10, and 50: (**a**) pure linear chain; (**b**) pure ring chain.

To interpret the intrinsic reason for the competitive adsorption transition occurred in bidisperse case, we focus on the monodisperse matrix of pure ring or linear chain. At the strong polymer-NP interaction, chain stiffness $k_{b}$ is also increased from 0 to 50. The different polymer conformations for pure linear and ring chain are presented in the snapshots of Figure 6a,b, respectively. For pure linear chain, we can obtain a general picture of local packing of linear chain in polymer-based nanocomposites, in which the linear chain is classified as the sequences of bridge adsorption, chiral helical adsorption, and tangent adsorption. In contrast, the ring chain shows a sequence of conformations of double bridges adsorption, coexistence of double bridges and orbital adsorption, and only orbital adsorption. In fact, this finding provides a direct support for understanding the competitive adsorption transition occurred in bidisperse case.

**Figure 7:**
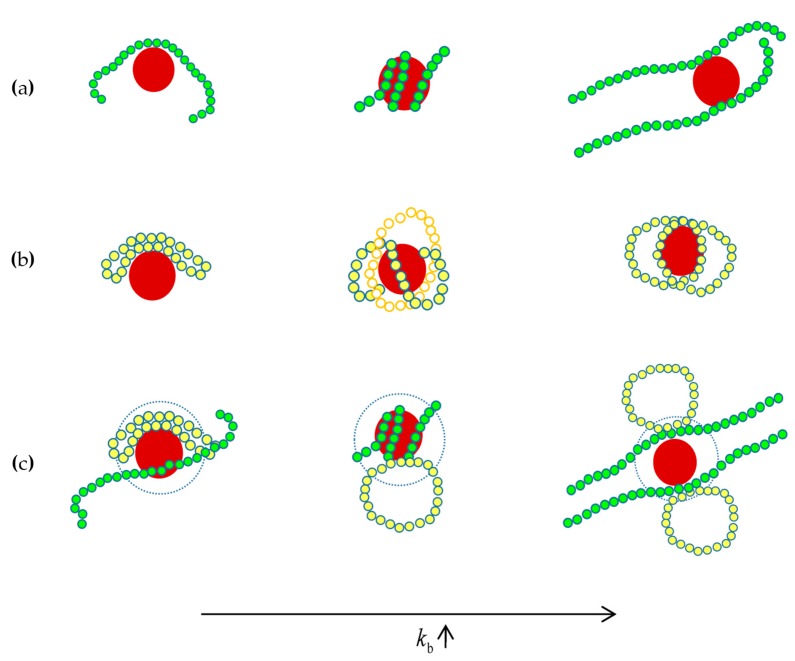
Schematic diagram of nanocomposites conformation at a strong attraction: (**a**) linear chain, (**b**) ring chain, (**c**) mixture of linear chain and ring chain.

The schematic diagram shown in Figure 7 offers a clear view for the local packing of monodisperse and bidisperse polymer matrix at the polymer-NP interface. As known by above, the “middle” adsorption mechanism dominates the polymer local packing in the interface under the strong polymer-NP interactions. Let us focus on the cartoons of Figure 7c. As the chain stiffness is weak, i.e., flexible chain, double bridge conformation of ring chain is superior to the single bridge packing of linear chain, illustrating “ring chain in majority” appeared in phase diagram of Figure 2. With the increase of chain stiffness, i.e., semiflexible chain, the helical structure of linear chain is benefit for wrapping the whole surface of NP, and crowding ring chain out of interface region. This finding can reveal the preferential transition from “ring chain in majority” to “linear chain in majority” occurred in phase diagram of Figure 2. Finally, as rigid-like chain, 3D tangent conformation of linear chain is absolute precedence over the 2D orbital plane structure of rigid-like ring, exactly explaining the absolute dominant adsorption state of f≈0 shown in Figure 3. Our work may provide a theoretical guidance for understanding the forming mechanisms of the conformations differences between linear chain and ring chain. Rcently, Iwamoto et al. investigated the conformations of ring plystyrenes by SANS and concluded Flory’s exponent ν in $R_{g}=N^{\nu }$ for rings may not be constant but rather show molecular weight dependence due to their topological constraint [41].

## 4. Conclusions

In conclusion, we investigated the local packing and competitive adsorption behavior of bidisperse matrix with ring and linear polymer chain using coarse-grained molecular dynamics simulations. It is found that for the weak polymer-NP interaction, ring chain are slightly preferred to occupy the interfacial region than linear chain, due to its closed topology structure and regardless of chain stiffness. While for the strong polymer-NP interaction, the “middle” adsorption mechanism dominates the polymer local packing in the interface, and the selective adsorption behavior of bidisperse matrix is sensitive to the chain stiffness. For flexible polymer chain, double bridge conformation of ring chain is superior to the single bridge packing from linear chain, resulting in “ring chain in majority”. As increasing chain stiffness, a ring-to-linear selective adsorption transition occurs. The semiflexible linear chain squeezes ring chain out of the interfacial region by forming a helical structure and wrapping tightly the surface of NP, while semiflexible ring chains still have the configurations of double bridge or 2D plane orbit. Further increasing stiffness to rigid-like chain, “linear chain in majority” selective adsorption behavior becomes more dramatic, in which 3D tangent conformation of linear chain is absolutely prior to the 2D plane orbital structure of ring chain. In addition, the monodisperse of pure ring or linear chain is also considered to explain the ring-to-linear selective adsorption transition with increasing chain stiffness. Besides, other topology structure e.g., star-like or dendritic chain will be the next step toward this issue that the effect of topology structure on adsorption behavior occurred at the polymer–NP interface.
